# Serotonin Impairment in CSF of PD Patients, without an Apparent Clinical Counterpart

**DOI:** 10.1371/journal.pone.0101763

**Published:** 2014-07-18

**Authors:** Enrica Olivola, Mariangela Pierantozzi, Paola Imbriani, Claudio Liguori, Mario Stampanoni Bassi, Marco Conti, Vincenza D′Angelo, Nicola Biagio Mercuri, Alessandro Stefani

**Affiliations:** 1 Department of Systems Medicine c/o Movement Disorder Centre, University of Tor Vergata, Rome, Italy; 2 IRCCS Fondazione S. Lucia, Rome, Italy; 3 Department of Systems Medicine c/o Neurophysiopathology Unit, University of Tor Vergata, Rome, Italy; INSERM/CNRS, France

## Abstract

In Parkinson's disease (PD), several studies have detected an impaired serotonin (5-HT) pathway, likely affecting both motor and non-motor domains. However, the precise impact of 5-HT impairment is far from established. Here, we have used a HPLC chromatographic method, in a homogenous cohort (n = 35) of non fluctuating, non dyskinetic PD patients, to assess the concentration of 5-HT and its metabolite 5-HIAA in peripheral cerebrospinal fluid (CSF) obtained from lumbar puncture (LP). LP was performed following three days of therapy withdrawal, in order to vanish the effects of prolonged released dopamine agonists (DA), and in absence of any serotonergic agent. The PD patient group showed a significantly reduced CSF level of both 5-HT and 5-HIAA compared to either age-matched control subjects (n = 18), or Alzheimer's disease patients (n = 20). However, no correlation emerged between 5-HT/5-HIAA concentrations and UPDRS-III (r = −0.12), disease duration (r = −0.1), age (r = −0.27) and MMSE (r = 0.11). Intriguingly, low CSF 5-HT levels did not differ for gender or for motor phenotype (in terms of non-tremor dominant subtype and tremor dominant subtype). Further, low CSF 5-HT levels did not correlate with the presence of depression, apathy or sleep disturbance. Our findings support the contention that 5-HT impairment is a cardinal feature of stable PD, probably representing a hallmark of diffuse Lewy bodies deposition in the brainstem. However, clinical relevance remains uncertain. Given these findings, an add-on therapy with serotonergic agents seems questionable in PD patients, or should be individually tailored, unless severe depression is present.

## Introduction

Parkinson's disease (PD) represents a multisystem disorder affecting several neurotransmitter pathways. According to Braak's staging of Lewy body deposition, the pathological changes underlying PD proceeds from the olfactory and the dorsal motor nucleus vagus to the midbrain, including the serotonergic raphe nuclei, which undergo an extensive degeneration [1].

Further, several studies, conducted in rodent or primate's PD models, have focused on the role of different serotonin (5-HT) receptor subtypes in either motor or non motor disease manifestations and ignited investigational trials on promising 5-HT ligands [2].

Yet, the influence exerted by 5-HT impairment in PD remains unclear. In the previous decades, a large amount of clinical studies on PD correlated the 5-HT impairment to depression [3], [4]. More recently, it has been proposed that serotonergic dysfunction may contribute also to the occurrence of relatively dopamine-resistant PD motor signs, including the development and the severity of postural and rest tremor [5] or the unmasking of L-DOPA-induced dyskinesia [6].

The occurrence and the extent of 5-HT impairment may be diagnoses through different techniques. A decrease of striatal 5-HT, its metabolite 5-hydroxyindolacetic acid (5-HIAA), 5-HT transporter and tryptophan hydroxylase, was documented by HPLC studies carried out in postmortem PD brains [7]. A more recent postmortem study [8] has shown a serotonergic hyper-innervation in the striatum, with the detection of dopamine in serotonergic varicose fibers. These findings may be interpreted as a compensatory mechanism for the lost function of dopaminergic neurons, due to the ability of 5-HT neurons to convert L-DOPA to dopamine [9].

A 5-HT dysfunction has been inferred also by *in vivo* functional imaging studies. An overall decrease of the 5-HT transporter SERT binding levels is detected in orbitofrontal cortex, caudate, putamen and midbrain of clinically advanced, non-depressed PD patients [10], albeit higher degree of impairment seems to occur in severe parkinsonism or Lewi Bodies Disorders [11]. Imaging studies with Position Emission Tomography (PET) inferred that the density of 5-HT transporters declines in the striatum of PD patients in a stage-dependent manner [12] whilst a global reduction of presynaptic serotonergic terminals occurs, however without a clear correlation with disease duration, motor disability and chronic exposure to dopamine replacement therapy [13].

An alternative approach utilizes biochemical markers, by collecting samples from cerebrospinal fluid (CSF) [14]–[16]. So far, also HPLC analysis has provided inconclusive results; (for instance, some reports indicated a reduced CSF concentration of 5-HIAA mostly in Multiple System Atrophy patients, but not in PD) [17].

Here, we have reconsidered this approach and determined CSF concentration of 5-HT and its main metabolite 5-HIAA in a cohort of relatively homogeneous PD patients that underwent lumbar puncture (LP) and a *brief* pharmacological washout. Our aim was to answer to two major open questions: are 5-HT levels decreased in PD? And, additionally, is there a correlation between serotonergic dysfunction and the occurrence of non-motor symptoms such as depression in PD?

## Materials and Methods

The study was approved by local ethical committee, the “Comitato Etico Indipendente”, Fondazione Policlinico Tor Vergata (Viale Oxford, 81, 00143, Rome Italy). Further, partecipants and their main caregiver (this applies to AD patients) have provided their written consent to participate in this study.

### Patients

Eligible PD patients had a diagnosis of idiopathic PD according to the UK Parkinson's Disease Society Brain Bank criteria, and a PD severity, assessed by the Hoehn and Yahr (H&Y) stage, ranging between 1 and 3. Patients also had disease duration between 12 and 60 months, were under stable anti-parkinsonian therapy, and showed no motor fluctuations or wearing off phenomenon. An age- and gender-matched healthy subject control group (affected by other neurological disorders – OND -) and a cohort of Alzheimer's Disease (AD) patients were included in the study (Table 1).

**Table 1:** It provides the mean epidemiologic features.

	PD	OND	AD
N	35	18	20
Male/female ratio	19/16	9/9	9/11
Age	66.53±4.22	67.09±4.08	69.38±3.01
MMSE	26.7±1.2	27.9±0.9	21.1±2.64
UPDRS part III	25.4±9.6	NA	NA
Disease duration, months	37.1±11.4	NA	21.6±10.8
Hoehn & Yahr Stage	1.9±0.5	NA	NA

No significant difference, for age and gender, between PD, OND and AD.

In PD and control group exclusion criteria were: cognitive impairment (Mini Mental State Examination MMSE <24), systemic diseases and use of serotonergic medications. In particular, none of our subjects was under selective serotonin reuptake inhibitors (SSRI) or tricyclic antidepressants (TCA) at time of LP or even in the three months preceding LP; further, only 4 out of the whole cohort had experienced, in previous years, a brief therapy with citalopram or escitalopram (and none was taking mirtazapine, whose possible binding to 5-HT1A receptors would represent a bias) [18]. At the time of enrollment, PD patients clinical motor impairment was quantified by the motor examination section of the Unified Parkinson's Disease Rating Scale (UPDRS-III) at the practically defined “off condition”, taken in the early morning before starting the daily pharmacological therapy. Depression was investigated through the Diagnostic and Statistical Manual of Mental Disease (DSM-IV) and the Beck Depression Inventory (BDI cut off for depression >13); apathy was investigated using the Apathy Evaluation Scale (AES), patient-rated version, with a score ≥38 considered positive for apathy. Nocturnal sleep disturbances were assessed using the Pittsburgh Sleep Quality Index (PSQI), where a score of >5 indicated significant nocturnal sleep disturbance.

As stated, informed consent was obtained from all participants or caregiver (for AD patients), and the local Committee approved the study.

### Collection and analysis of CSF samples

CSF was obtained by LP performed in the lateral decubitus position, between 8 and 9 a.m. As concern PD patients, LP was executed after three-day dopaminergic therapy withdrawal. This washout was chosen since CAPIT does not allow to vanishing in full the effect of prolonged released dopamine-agonists (DA) (on the other hand, 5 or more days were considered unethical by our Ethical Committee).

To minimize the rostro-caudal gradient, LP was performed at distinct interspaces according to patients' height (L4-L5, height 160–170 cm; L3–L4, height 170–180 cm; L2–L3, >180 cm) [19].

The first 4 ml sample was used for routine analyses. Then a sample (2 ml) was collected, protected from light, centrifuged at 3000 x g for 10 min at 4°C to remove cells and other insoluble material and acidified by adding 10 µL of perchloric acid and immediately frozen at −80°C until analysis. Determination of monoamines levels in CSF was determined by high-performance liquid chromatography (HPLC) analysis coupled with electrochemical detection. The chromatographic method utilized (Alexys monoamine analyzer-Decade II) is optimised for small sample volumes (3 or 5 µL injection) with a detection limit below 50 pmol/L for the biogenic amines. By means of a dual loop 10-port valve a single injection is loaded on two LC-flow paths simultaneously. For separation of the components of interest, a suitable combination of column (type Antec ALF -115 column, 150×1.0 mm ID, 3 µm C18 and ALF-105 column, 50×1.00 mm ID 3 µm C18). Linear calibration graphs of the peak-area ratio of each compound to the internal standard versus the concentration of the compound studied were constructed.

### Statistical analysis

Demographic data were assessed by Kruskal-Wallis test. Biochemical data were analyzed by performing Kruskal-Wallis, with the Dunn's post hoc test (PD vs. OND vs. AD). Correlations between metabolites were assessed by Spearman's correlation. For each variable, median and mean are reported in tables.

## Results

35 subjects with idiopathic PD, 18 control subjects and 20 AD patients were recruited for this study (Table 1). The control group (OND) included patients affected by peripheral nervous system disturbances (i.e neuropathies, and mono-radiculopathies studied for defining possible surgical indication) plus 3 suffering from cephalalgia. None of them show any impairment attributable to neurodegenerative diseases, (neither at time of LP, nor during follow-up). No statistically significant differences in age and distribution of male/female ratios were found by comparing the groups.

**Figure 1:**
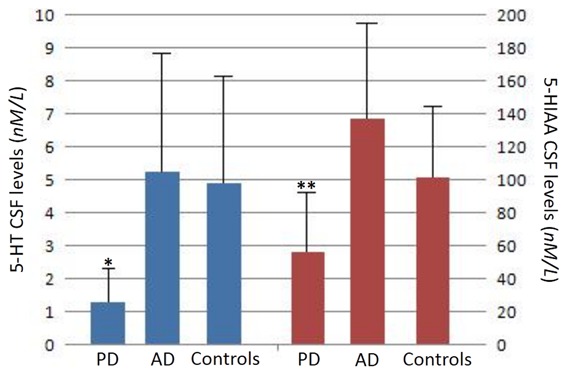
The histograms report the mean (and SD) CSF concentration for 5-HT (left, blue) and 5-HIAA (right, red) in the three studied populations. Note the different scaling in the y axes. PD cohort showed significantly lower levels (* p<0.05; ** p<0.005).

PD group showed a significant reduction of both CSF 5-HT and 5-HIAA levels, compared to OND group (p = 0.015 and p = 0.002) as well as to AD group (p = 0.02 and p = 0.001) (Fig.1). Moreover, 5-HT and 5-HIAA did not differ between AD and OND groups (Fig. 1).

**Figure 2:**
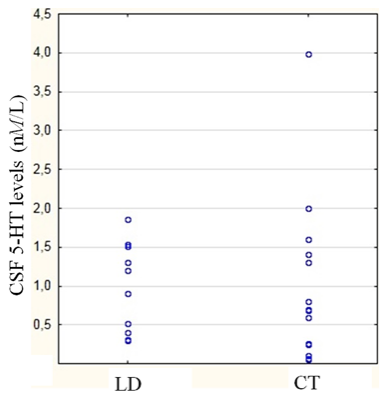
The graph shows the individual 5-HT concentration levels, comparing PD patients under LD alone (LD, n = 10) and DA plus LD (CT, combined therapy, n = 15). The difference is not significant. Not included, due to exiguity of the samples, the values of *denovo* (n = 6) and patients under DA alone (n = 4). To note, any therapy regards the period preceding the 3-days washout implicit to the study.

In order to ascertain, besides the brief washout, whether chronic treatment with DA exerted a profound influence onto CSF 5-HT concentrations, a comparison was done between PD patients exclusively under LD with PD patients on therapeutic cocktails including LD plus DA; as shown in Fig. 2, no significant difference emerged (0.98 nM+0.57 in the LD group; 1.18 nM+0.62 in LD + DA group, p = 0.95). (*de novo* patients and patients under DA alone were not examined in light of their exiguity – n = 6 the de novo, n = 4 for DA alone -).

Of note, therapeutic history included DA in 19 PD patients (4 patients under DA alone and 15 patients under DA plus LD); these patients were similarly distributed in terms of gender, motor phenotype, presence or absence of depression.

No significant correlation emerged between CSF 5-HT and 5-HIAA concentration and UPDRS-III (r = −0.12), disease duration (r = −0.1), age (r = −0.27) and MMSE (r = 0.11). In addition, 5-HT CSF levels did not correlate with BDI (r = 0,12). As expected, a statistically significant positive correlation was found between UPDRS-III and disease duration (r = 0.71).

Finally, we examined the PD patients stratified by different parameters. As shown in Table 2, 5-HT and 5-HIAA levels were not significantly different for gender, PD phenotype (TD vs non-TD), and the presence or absence of depression, apathy, and sleep disturbances.

**Table 2:** The table shows the lack of significant difference in CSF 5-HT (†) and 5-HIAA (‡) levels, when PD patients are sub-divided by gender, disease phenotype, and selected non-motor features.

Gender (M/F)	19/16	^†^ P>0.05	^‡^ P>0.05
TD vs non-TD	13 vs 22	**^†^** P>0.05	**^‡^** P>0.05
Depression (BDI>13)	Yes/No (14/21)	**^†^** P>0.05	**^‡^** P>0.05
Apathy (AES≥38)	Yes/No (9/26)	**^†^** P>0.05	**^‡^** P>0.05
Sleep Disturbances (PSQI>5)	Yes/No (11/24)	**^†^** P>0.05	**^‡^** P>0.05
Hoehn & Yahr: ≤1,5/>1,5	17/18	**^†^** P>0.05	**^‡^** P>0.05

BDI  =  Beck Depression Inventory; AES  =  Apathy Evaluation Scale PSQI  =  Pittsburgh Sleep Quality Index.

## Discussion

This investigation clarifies that a significant reduction of CSF 5-HT and 5-HIAA levels occurs in PD patients, irrespectively to their clinical motor and non-motor profile, as well as to their disease duration and staging, at least in non fluctuating ones. In fact, in the present study we excluded fluctuating or extremely advanced PD patients, in order to avoid potential biases due to the complex neurotransmitter and circuitry changes that are postulated to underlie more advanced disease stages (including dyskinesia).

The relevance of our finding is strengthened by the observation that a coeval cohort of AD patients did not show similar impairment of serotonergic system, enforcing the idea that the decrease of 5-HT and 5-HIAA levels found in PD patients is not related to a-specific mechanisms of neurodegeneration, rather representing a specific feature of the synucleopathy. Indeed, the last decade was dominated by a growing body of evidence suggesting that PD is not only a motor dopaminergic disease, but also a complex multisystem disorder. One of the more inquired non-DA system is the endogenous serotonergic pathway. Consistently, here, a significant reduction in CSF 5-HT emerged.

However, what is the clinical counterpart of this finding? In light of our data, a straightforward identification of its clinical relevance is difficult. In particular, CSF levels show no correlation between depression and 5-HT and 5-HIAA reduction, as reported in de-novo PD patients [14]. The limited sample, examined so far, probably impeded to find a clear correlation of the female gender (in which frequency of depression and anxiety seems significantly higher) [20] with a specific CSF profile of 5-HT and 5-HIAA.Yet, our findings are in agreement with a recent PET study proving a global reduction of pre-synaptic serotonergic terminals in PD without a clear correlation with disease duration, motor disability and chronic exposure to dopamine replacement therapy [13]. The hypothetical strict correlation linking 5-HT and depression in PD is currently under scrutiny, since it might be biased by several confounding factors such as different regimen of therapies or unreliability of subjective scoring. Not surprisingly, studies that combined imaging with a stable ongoing therapeutic regimen have suggested that the mere optimization of dopaminergic-centered therapy (for instance by DA) may be able to recover depression in PD patients [21].

Nevertheless, the lack of correlation between depression and 5-HT content, as presently showed, does not exclude the possibility that more subtle psychopathologies are influenced by serotonergic deficit in PD. It is possible that in a larger sample of patients, undergoing a prolonged follow-up study, low levels of 5-HT would correlate with specific sub-items related to unconventional aspect of non-motor symptoms well beyond those testable through depression or apathy rating scales, including BDI, HSD or AES. Coping strategies, for instance, are largely impaired in PD patients whereas reduced compliance and lack of adhesion to therapy is largely recognized despite consistent L-dopa-therapy and STN-DBS treatment [22].

Although our CSF data indicate that serotonergic tone is indeed compromised in PD, we are aware that there are some limitations of this study. First, it offers a “peripheral” assessment of serotonergic dysfunction, by analyzing 5-HT and its metabolite from “lumbar CSF”; nevertheless, studies based on the measurement of serotonin markers directly from brain did not describe rather different patterns [7]. Besides, the technique of extraction and measurement of 5-HT levels used here is rather sensitive, and the results obtained are not at odds with those found in other studies [14]–[16].

Furthermore, the acquisition of CSF 5-HT concentration, *per se*, does not provide a full vision on the endogenous states of specific 5-HT receptor subtypes. For instance, several disease models have shown strong evidence in favor of an up-regulation of 5-HT1A receptors in combination with 5-HT reduction [23], [24]. Of course, studies employing multiple approaches, including PET, might contribute answering this issue.

Moreover, we are aware that 5-HT neurons, that are able to convert exogenous L-DOPA to dopamine, are a major site of dopamine release throughout the brain. It has been clearly documented how the substitution of 5-HT by newly synthesized dopamine, in 5-HT neurons, leads to acute and chronic compensatory-like alteration of 5-HT release and metabolism [25]. Yet, the importance of this “compensatory endogenous mechanism”, in our patients, seems modest when considering the lack of correlation between CSF 5-HT and disease duration and the similar 5-HT/5-HIAA ratio amongst the studied populations. Conversely, the investigation on dopamine metabolites (3,4-dihydroxyphenylacetic acid – DOPAC - and homovanilic acid - HVA -) in PD patients with similar [26] or more advanced [27] disease stages indicated a subtle but clear increase of dopamine turnover.

Longitudinal studies, with repeated LP in the same subject, might clarify better whether the evolution of the 5-HT impairment correlates with disease progression rate.

Incidentally, our study has shown that AD patients do not manifest a clear-cut impairment of endogenous 5-HT, at least when evaluated through the CSF analysis here utilized. Of course, this research line deserves supplemental investigation, given the probable correlation of an altered 5-HT/5-HIAA ratio and specific psycho-pathological traits (mostly in frontotemporal dementia or AD featuring aggressive behaviors) [28].

For the time being, the low CSF 5-HT content detected in PD patients may represent the biochemical demonstration of the involvement of extra-DA pathways, but it is still in search of a clinical relevance.
